# Dry Sliding Tribological Behaviors of Electrodeposited Ni-GO/SiC Composite Coating on the 2218 Aluminum Alloy

**DOI:** 10.3390/ma15082834

**Published:** 2022-04-12

**Authors:** Yutao Yan, Lifeng Lu, Yuqiu Huo

**Affiliations:** 1 School of Mechanical Engineering and Automation, Northeastern University, Shenyang 110819, China; 2070181@stu.neu.edu.cn; 2 Department of Chemistry, College of Science, Northeastern University, Shenyang 110819, China

**Keywords:** composite coating, electrodeposition, friction coefficient, wear rate, wear mechanism

## Abstract

Electrodeposition has attracted tremendous interest in functional coatings due to its advantages of high efficiency, inexpensiveness and ease of implementation. In this work, nickel graphene oxide (Ni-GO), nickel silicon carbide (Ni-SiC) and nickel graphene oxide/silicon carbide (Ni-GO/SiC) composite coatings were electrodeposited on the 2218 aluminum alloy (2218AlA) substrate. The microstructure, microhardness, bonding strength and tribological behaviors of the composite coatings were carried out. According to the results obtained, the composite coatings were dense and compact, with no visible defects and microcracks, and well bonded to 2218AlA substrate. The microhardness of composite coatings was significantly increased compared to that of the 2218AlA substrate. The microhardness of Ni-SiC composite coating was the highest, reaching 3.14 times that of the 2218AlA substrate. The friction response time, friction coefficient and wear rate of the composite coatings were obviously lower. For the Ni-GO composite coating, the average friction coefficient is the smallest at 45.35% of the 2218AlA substrate, while the wear rate is the smallest at 46.97% of the 2218AlA substrate. However, the comprehensive tribological performances of the Ni-GO/SiC composite coating were superior. The abrasive and adhesive wear were the main wear mechanisms of composite coatings, but the degree of damage was different.

## 1. Introduction

The surface material of mechanical parts is continuously removed due to wear, reducing the service life and leading to a significant economy and energy consumption [1,2]. The application of coating with good tribological performances is a good way to increase the friction reduction and wear resistance; the coatings are usually used to decrease friction and wear by forming a protective layer between friction interfaces [1,3]. Commonly used coating techniques include laser cladding [4], plasma spraying [5], electrodeposition [6], vapor deposition [7,8] and sol-gel [9], etc. Among them, electrodeposition technology has aroused great interest in the synthesis of functional coatings because of its simplicity, ease of implementation, low cost, it does not require specialized equipment and ease of applying to the substrate of different shapes [1,10,11,12].

Graphene oxide (GO) is a two-dimensional nanomaterial with many functional groups, which has excellent tribological performances, good mechanical performances, high electrical conductivity and thermal performances [13,14,15]. Some of these functional groups of GO contribute to its co-deposition with metal ions, resulting in a coating with excellent wear and corrosion resistance. Singh et al. [16] prepared a Ni-GO composite coating by the pulsed electrodeposition. The results showed that the composite coating could effectively reduce the friction coefficient and wear rate and was superior to the individual electrodeposited Ni coating. It was reported by Tamilarasan et al. [17] that the Ni-P-rGO composite coating had a good adhesion, and improved the microhardness, friction reduction and antiwear of mild steel. In addition, the wear mechanism of the composite coating was explored. A homogeneous reinforced Ni-graphene composite coating was fabricated by an exfoliation technique for solving the agglomeration of graphene, and the mechanical performances and tribological behaviors of the composite coating were investigated. It was found that the addition of graphene could improve the microhardness and reduce the friction coefficient and wear rate [18,19]. Hdz-García et al. [20] prepared a Ni-rGO composite coating onto the surface of austenitic stainless steel by the sol-gel method and investigated its tribological behaviors. The results indicated that the reduction of coefficient of friction and wear rate was mainly attributed to the self-lubricating performances of rGO and the effect on the grain refinement of the composite coating. Zhang et al. [21] studied the friction and wear mechanisms of GO from atomistic insights and found that the increasing of antiwear of GO was attributed to the reduction of its epoxy groups. Likewise, tribological behaviors of composite coatings of GO coexisting with other particles have been carried out. The self-lubrication of GO had an important role in reducing friction coefficient and wear rate [14,22,23,24].

Composite coatings with high hardness ceramic particles are often used as an effective means of improving wear resistance. The SiC particles in the composite coatings can effectively enhance the hardness and wear resistance and reduce the friction coefficient [1,25]. Guo et al. [26] fabricated a Ni-SiC composite coating on TA15 alloy by electroplating technology and investigated its tribological performances. Compared with the TA15 alloy substrate, the composite coating showed higher microhardness and significant high-temperature friction reduction than the TA15 alloy substrate. Gyawali et al. [27,28] investigated the comprehensive performances of Ni-SiC composite coatings after the ultrasonic nanocrystal surface was modified and found that the microhardness, wear resistance, friction reduction and corrosion resistance were improved. Tailor et al. [29] studied mechanically alloyed 6061Al-SiC_p_ composite coating by plasma spraying. It was found that the composite coating had high bounding strength and microhardness, and the wear rate was 50% lower than that of the 6061Al. In general, the main reason for the enhanced wear resistance of SiC ceramic particles is that their addition increases the hardness of the composite coating.

In this work, the nickel graphene oxide (Ni-GO), nickel silicon carbide (Ni-SiC) and nickel graphene oxide/silicon carbide (Ni-GO/SiC) composite coatings with excellent tribological behaviors were fabricated on 2218 aluminum alloy (2218AlA) substrate by the electrodeposition technology. The surface morphologies, microhardness and bonding strength of the prepared composite coatings were assessed. The tribological behaviors were investigated using a dry sliding friction experiment. Then, the wear mechanism of the experimental samples was explored.

## 2. Experimental

### 2.1. Materials and Sample Preparation

Commercially 2218AlA is chosen as the substrate material in this study. Table 1 shows the chemical composition of the purchased 2218AlA. Graphene oxide (GO) nanosheets were prepared by our research team in the laboratory based on a modified Hummer’s method. The detailed characteristics can be found in Reference [30]. Silicon carbide (SiC) power was commercially purchased from Shanghai Chaowei Nanotech Co., Ltd., Shanghai, China (≥99.5%, an average size *d* = 600 nm, *ρ* = 1.52 g/cm^3^).

**Figure 1:**
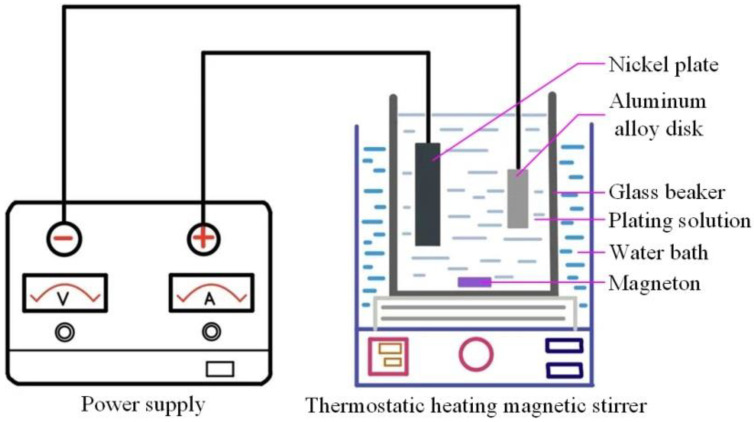
Schematic diagram of the composite coating preparation.

The 2218AlA was cut into the disk of the dimensions *ϕ*50 × 8 mm. The surfaces of each disk were gradually polished to 0.22 μm with the abrasive papers and then ultrasonically cleaned with deionized water for 5 min. For obtaining a good quality composite coating, it is necessary to pretreat the surface of the disks before the electrodeposition. The pretreatment process includes degreasing the disk surface using acetone, removing surface oxides, and surface activation by soaking in a mixed solution of 30 g/L Na_2_CO_3_, 30 g/L NaOH and 30 g/L Na_3_PO_4,_ and a zinc replacement treatment. After each procedure, the disks were ultrasonically rinsed with deionized water for 5 min. A pure nickel plate and the pretreatment disk were used as the anode and cathode for the electrodeposition process, respectively, and the distance between them was kept at 30 mm. The pH of the plating solution was adjusted to 3 using the citrate. In order to achieve the high-quality electrodeposition, the plating solution was stirred with a magnetic stirrer at 300 rpm for 8 h at room temperature. The samples of the composite coating were prepared in a 50 mL beaker filled with the plating solution. Table 2 shows the composition of the plating solution and the operating conditions. For the convenience of description, the prepared Ni-GO, Ni-SiC and Ni-GO/SiC samples are defined as S2, S3 and S4, respectively. The 2218AlA sample is defined as S1. The electrodeposition process for the preparation of the composite coatings is shown in Figure 1. This device mainly includes a DC regulated voltage and current power supply (WYK-5010, Yangzhou Jintong Eletronics Co., Ltd., Yangzhou, China) and a collector-type thermostatic heating magnetic stirrer (DF-101S, Gongyi Yuhua Instrument Co., Ltd., Zhenfzhou, China). During the electrodeposition process, the power supply ensures a stable current density. The thermostatic magnetic heating stirrer was used to maintain the bath temperature at 50 °C and perform continuous stirring.

### 2.2. Tribological Experiment

**Figure 2:**
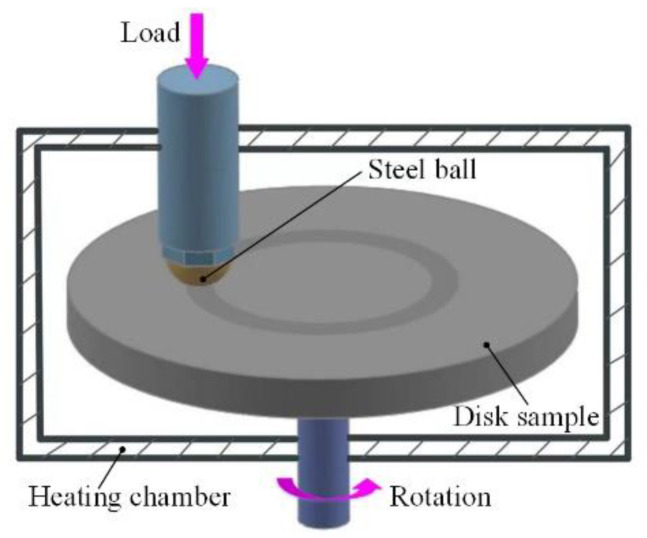
Schematic diagram of the rotating ball-on-disk contact configuration.

The tribometer (HT-1000, Zhongke Kaihua Technology Development Co., Ltd., Lanzhou, China) investigated the dry sliding tribological experiments with a rotating ball-on-disk configuration. The schematic diagram is shown in Figure 2. Commercial AISI 52100 steel balls with a diameter of 5 mm, a roughness of 0.05 μm and a hardness of 780 HV were used as the counterbody. All experiments were carried out in air for 10 min at 125 °C and a normal load of 2 N. The rotation speed and rotation radius were taken as 300 rpm and 8 mm, respectively. The friction coefficient was measured continuously and automatically by the tester. The samples were ultrasonically cleaned using deionized water at room temperature for 10 min before and after each experiment and then blown dry. The wear loss was obtained by the weighing method using an electronic balance with an accuracy of 0.1 mg (FA2204C, Shanghai Yueping Scientific Instrument Co., Ltd., Shanghai, China). The wear rate *R*_w_ was calculated by the formula *R*_w_ = Δ*M*/*FL*, where Δ*M* is the wear mass (mg), *F* is the applied normal load (N), *L* is the distance of sliding (m). Each sample was conducted for at least three experiments.

### 2.3. Characterization and Analysis Methods

The surface morphology of composite coating, cross-section microstructure and worn surface were analyzed by scanning electron microscopy (SEM, MIRA3, TESCAN, Czech Republic). The elemental analysis of composite coatings was carried out by energy dispersive spectroscopy (EDS). The three-dimensional morphologies of the worn surface were observed by a 3D optical microscope (DVM6, Germany). The microhardness of all samples was obtained using a digital Vickers hardness tester (THV-5, Beijing Time High Technology Co., Ltd., Beijing, China). In this case, a load of 200 g was applied within a dwell time of 15 s at room temperature. Given the microhardness distribution, each sample’s microhardness was determined using an average of at least five measurements in its different areas. A coating automatic scratch instrument (WS-2005, Zhongke Kaihua Technology Development Co., Ltd., Lanzhou, China) was employed to obtain the bonding strength of all composite coating samples by the scratch method. In this case, the diamond indenter with a 120° conical shape and a tip radius of 200 μm was employed. The test conditions are as follows: the loading rate is set to 20 N/min, and the scratch rate is set to 6 mm/min. According to Reference [31], the bonding strength of each composite coating sample was obtained. The average of at least three times was determined.

## 3. Results and Discussion

### 3.1. Characterization of Composite Coating

**Figure 3:**
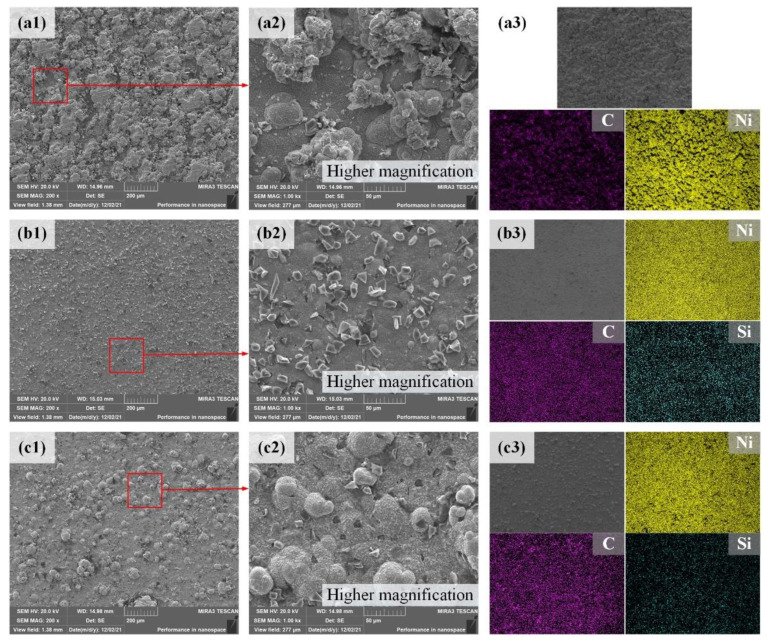
SEM images and EDS mapping for composite coatings. (**a1**–**a3**) S2 sample; (**b1**–**b3**) S3 sample; (**c1**–**c3**) S4 sample.

Figure 3 shows the SEM images and EDS mapping analysis of the composite coating surfaces. The surface of the S2 sample is relatively rough and has a surface morphology similar to cauliflower. But the overall distribution is relatively homogeneous. The Ni grains are rounded, fine and evenly distributed. The ions in the plating solution quickly penetrated and accumulated and then formed a few nodulations on the composite coating surface, as shown in Figure 3(a1,a2). The GO nanosheets can supply more Ni ions nucleation sites due to their large specific surface area. The Ni grains are small and regular during the electrodeposition process due to the GO nanosheets hindering the Ni ions’ growth process. Meanwhile, the synchronous exfoliation and deposition can effectively reduce the agglomeration of the GO nanosheets [18,19]. The EDS analysis of the S2 sample surface in Figure 3(a3) shows that the distribution of the Ni and the C elements is very uniform. Compared with the composition of the 2218AlA substrate in Table 1, S2 sample surface is enriched with a large amount of the Ni and the C elements with atomic percentages are 29.52% and 65.30%, respectively. These indicate that the evenly composite coating of the Ni and the GO co-deposited on the 2218AlA substrate is achieved. The dispersion of GO nanosheets in the plating solution is very good. The surface of S3 sample in Figure 3(b1,b2) show smoother and more intact. The SiC particles are evenly and irregularly distributed in the composite coating with light agglomeration. Compared with the S2 sample, the Ni crystals are finer. It appears that the addition of SiC particles can effectively hinder the Ni grains growth and generated a smoother surface [27,28]. The surface of composite coating is uniformly enriched with a large amount of the Ni, C and the Si elements, indicating that a uniform Ni-SiC composite coating is obtained on the 2218AlA substrate, as illustrated in Figure 3(b3). The atomic percentages of Ni, C and Si elements are 25.16%, 57.72% and 9.85%, respectively. The surface of S4 sample is smoother than that of the S2 sample and rougher that of the S3 sample. It is observed that the surface is uniformly distributed with a hummocky morphology of different sizes, as shown in Figure 3(c1,c2). It is believed that during the electrodeposition process, the SiC particles reduce the Van der Waals forces between the GO layers, enhance the stability of the GO nanosheets and hinder the accumulation of the GO nanosheets due to the reduction of the nodule size. Meanwhile, the SiC particles are netted by the GO nanosheets. The S4 sample surface is uniformly enriched with a large amount of Ni, C and Si elements, indicating that a homogeneous Ni-GO/SiC composite coating is obtained on the 2218AlA substrate, as illustrated in Figure 3(c3). The atomic percentages of Ni, C and Si elements are 33.02%, 57.86% and 8.26%, respectively. These indicate that the GO nanosheets and SiC particles can be well dispersed in the plating solution.

**Figure 4:**
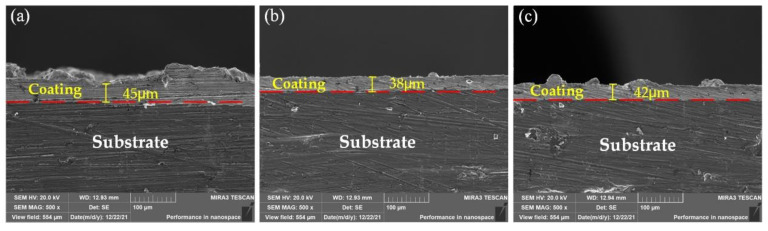
SEM images for the cross-section. (**a**) S2 sample; (**b**) S3 sample; (**c**) S4 sample.

The SEM images of the cross-section of the composite coatings are shown in Figure 4. It can be observed that the Ni-GO, Ni-SiC and Ni-GO/SiC composites are uniformly coated on the 2218AlA substrates. No visible defects and micro-cracks are observed at the interface, and the composite coatings have a continuous, compact and intact structure. This indicates that all composite coatings have a good bond with the 2218AlA substrate. The surface of the S2 sample is rougher, as illustrated in Figure 4a. The S3 sample has a smoother surface, as shown in Figure 4b. Nevertheless, the S4 sample is a relatively smooth surface with protruding structure, as depicted in Figure 4c. These are consistent with the SEM image morphologies of composite coating surface corresponding to Figure 3. The thicknesses of composite coatings of S2, S3 and S4 samples are about 45 μm, 38 μm and 42 μm, respectively. It indicates that the thickness of the composite coating is dependent on the electrodeposition rate.

### 3.2. Tribological Behaviors

**Figure 5:**
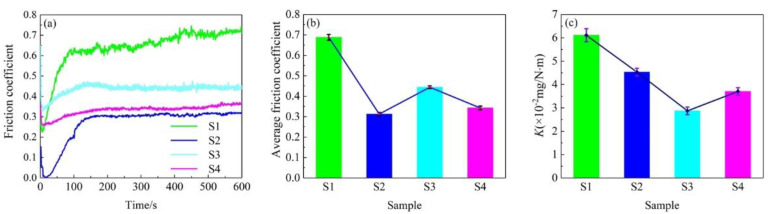
Variation of friction coefficient and wear rate. (**a**) Friction coefficient vs. time; (**b**) Average friction coefficient; (**c**) wear rate.

The variation of friction coefficient and wear rate is shown in Figure 5. It is clearly seen that the friction coefficient of S1, S2, S3 and S4 samples gradually increases at the beginning of the experiment and then fluctuates in a stable interval. However, the response time to reach a steady friction state is significantly different for each sample, as shown in Figure 5a. The response time is about 420 s for the S1 sample. Nevertheless, the response time of S2, S3 and S4 samples is obviously reduced. For instance, the response time of the S2 sample decreased by nearly 64.29% compared to the S1 sample, which is approximately 150 s. The response time of S3 and S4 samples is approximately 220 s and 180 s, respectively. Compared with the S1 sample, the response time of S3 and S4 samples are reduced by 47.62% and 57.14%, respectively. The response time of the S4 sample is greater than the S2 sample but less than the S3 sample. In other words, the running-in characteristic of the S4 sample is superior to that of the S3 sample and inferior to that of the S2 sample. It is very important that the friction pairs quickly reach the stable friction state, which can immensely decrease the running-in time and energy consumption and enhance working performances. As illustrated in Figure 5b, it is found that the average friction coefficient of composite coating samples is less than that of the S1 sample. The average friction coefficient of the S1 sample is 0.688, indicating its poor friction reduction ability. However, the average friction coefficient of the S2 sample is the minimum of 0.312, which is only 45.35% of that of the S1 sample, indicating that the S2 sample has the best friction reduction property. The average friction coefficient of S3 and S4 samples are reduced by 35.47% and 50.29% compared to the S1 sample, respectively. It is noteworthy that the average friction coefficient of the S4 sample is 1.10 times that of the S2 sample and 0.77 times that of the S3 sample. In other words, the friction reduction performance of the S4 sample is between S2 and S3 samples. As shown in Figure 5c, it can be seen that the wear rate of the samples is distinctly different. Compared with the S1 sample, the wear rates of all composite coatings are decreased. The wear rate of the S1 sample is the maximum of 6.11 mg/N·m, while the wear rate of the S3 sample is the minimum at only 46.97% of the S1 sample. Meanwhile, the wear rate of S2 and S4 samples are reduced by 25.86% and 39.44% compared to the S1 sample, respectively. In other words, the wear rate of the S4 sample is better than that of the S2 sample but inferior to that of the S3 sample. Based on the above analysis, it is found that the wear resistance and friction reduction performances of a single coating are not consistent. In this work, the S2 sample has the best friction reduction performance, while the wear resistance of S3 sample is the best. The other researchers have reported that the addition of GO or/and SiC can effectually improve the tribological performances of composite coatings [18,19,27,32]. It is possible to conclude that GO nanosheets’ improved friction reduction performance may be attributed to their excellent lubricating performances and the formation of a good protective layer of tribological film between the interfaces [19,33,34]. The silica oxides can be formed on the worn surface on account of frictional chemical reactions during the dry sliding friction experiment, which improved the friction reduction performance of composite coatings containing SiC particles [35]. It is well known that the surface hardness of a material is inversely proportional to the wear rate according to the widely used Archard’s wear model [36,37]. Therefore, the improved wear resistance of the composite coatings may depend mainly on the increase of their surface microhardness. In general, the friction coefficient and wear rate values are also connected with the coverage and uniformity of distribution of GO nanosheets or/and SiC particles on the contact surface. As analyzed above, it can be found that the tribological performances of the S4 sample are superior if both friction reduction and wear resistance performances are considered.

**Figure 6:**
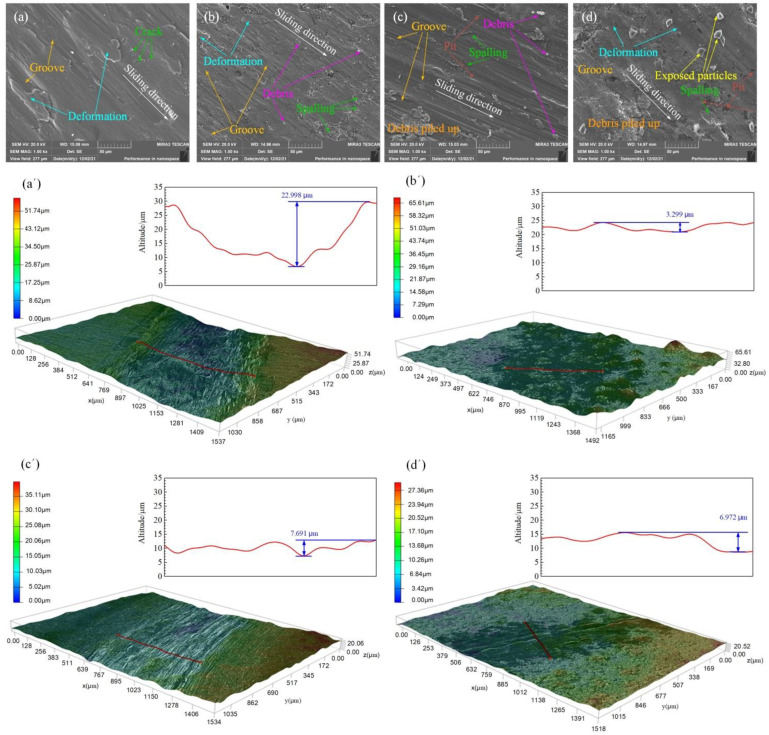
SEM images and 3D morphologies of the worn surface. (**a**,**a’**) S1 sample; (**b**,**b’**) S2 sample; (**c**,**c’**) S3 sample; (**d**,**d’**) S4 sample.

For further comprehension of the wear mechanism, Figure 6 shows the worn surfaces’ SEM images and 3D morphologies. The worn surface of the S1 sample exhibits a water wavy form mark along the sliding direction. It can be observed that there is severe plastic deformation and plastic flow due to the low microhardness of the 2218AlA and irregular wide and deep grooves owning to the plowing effect of hard bulging of the counterbody and wear debris. Simultaneously, there are a small number of microcracks and tear marks. The worn surface is rough, and the wear depth is deeper at 22.998 μm, as shown in Figure 6(a,a’). It is suggested that severe adhesive wear, abrasive wear and mild fatigue wear are the dominant wear mechanism of the S1 sample. The worn surface of the S2 sample shows an overall smear-like morphological feature. It can be seen that fine and very shallow grooves are uniformly distributed in the direction approximately perpendicular to the sliding direction, and these grooves are not continuous in the sliding direction. At the same time, there are the wear marks of plastic deformation, debris and flake-like spalling. However, the plastic deformation is slighter than that of the 2218AlA substrate. The worn surface is overall flatter and smoother. There are shallow wear marks with a wear depth of 3.299 μm, as illustrated in Figure 6(b,b’). During the continuous sliding friction, the bearing and lubricating performances of the GO nanosheets are fully exploited. The formation of the GO-enrich lubricant layer effectively filled the grooves of the contact surface, indicating that the composite coating exhibits good lubrication performances [19,38]. This should be the main reason for the lowest friction coefficient of the S2 sample, as illustrated in Figure 5b. Therefore, mild adhesive wear and abrasive wear are the main wear mechanism of the S2 sample. As shown in Figure 6(c,c’), the deeper and wider groove with a more even distribution can be seen on the S3 sample worn surface, which is continuous along the sliding direction. However, there is no obvious plastic deformation phenomenon due to the high microhardness. In addition, it can be observed that there is a debris of different sizes, flake-like spalling, local debris piled up and a small number of micropits. The worn surface is rougher than that of the S2 sample and flatter and smoother than that of the S1 sample. The wear depth of the S3 sample is 7.691 μm, which is 33.44% of the S1 sample and 2.33 times of the S2 sample. In the continuous sliding friction process, a small number of SiC particles are removed from the composite coating due to the extrusion and grinding of the counterbody and form third body abrasive particles between the friction interfaces, resulting in a small number of micropits and severe scratch marks on the worn surface. This should be the dominant reason for the severe abrasive wear of the S3 sample. A similar wear mechanism was also reported by Huang et al. [25]. Therefore, abrasive wear is the main wear mechanism of the S3 sample. The worn surface of the S4 sample exhibits relatively wide smear-like wear marks and discontinuous shallow grooves. Meantime, it can be observed that there is mild plastic deformation, micropits of different sizes, debris piled up, spalling and a small number of semi-exposed SiC particles. The worn surface is rougher than that of the S2 sample and smoother than that of the S3 sample. The wear depth is about 6.972 μm, which is 2.11 times the S2 sample and 87.58% of the S3 sample, as illustrated in Figure 6(d,d’). In this case, the result may be attributed to a combined effect of SiC particles shedding, scratching of third body SiC particles and the lubricating performance of the GO nanosheets and its scratch-filling effect. Therefore, the S4 sample may be considered superior from the viewpoint of the overall performance of friction reduction and wear resistance. The mild abrasive wear and adhesive wear are the main wear mechanisms of the S4 sample.

### 3.3. Mechanical Performances

**Figure 7:**
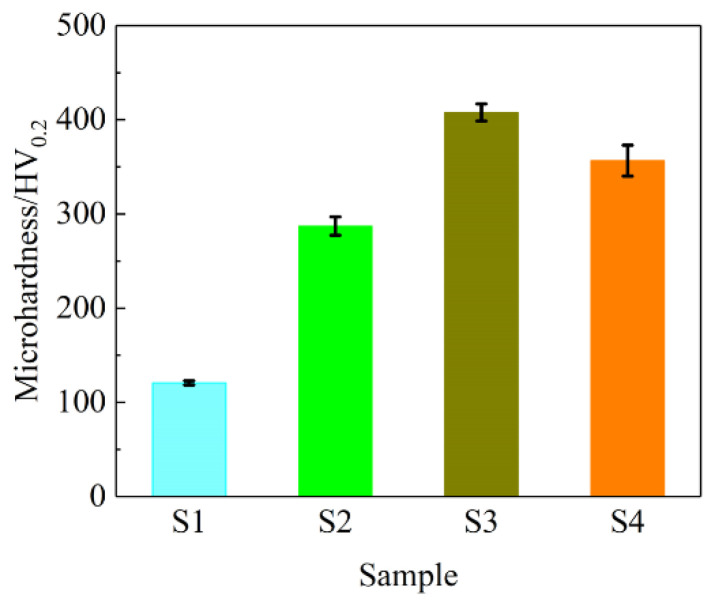
Microhardness of all samples.

In general, there is a close correlation between the surface hardness of a material and its tribological performances. The hardness is used to characterize the wear resistance, anti-scratch and cutting resistance [6,34,39,40]. In order to investigate the reasons for the good tribological behaviors of the composite coatings, the microhardness of all samples is measured. Figure 7 shows the microhardness of S1, S2, S3 and S4 samples. S1 sample has the smallest microhardness of 129.72 HV. Compared with the S1 sample, the microhardness of the composite coating samples is enhanced. The microhardness of the S3 sample has a maximum of 407.76 HV, which is 3.14 times that of the S1 sample and is in good agreement with that reported in the literature [25]. Such a trend of microhardness improvement was reported by Gyawali et al. [27,28]. Likewise, compared with the S1 sample, the microhardness of S2 and S4 samples are increased by 121.31% and 174.84%, respectively. It shows that the fabrication of electrodeposition composite coatings increases the microhardness of the S1 sample. The increase in microhardness of composite coating samples is mainly attributed to particle strengthening, grain refinement and finer structure appearance [41,42,43]. This increase in microhardness helps enhance the overall wear resistance of composite coatings compared to the S1 sample, which agrees well with the results in Figure 5c.

**Figure 8:**
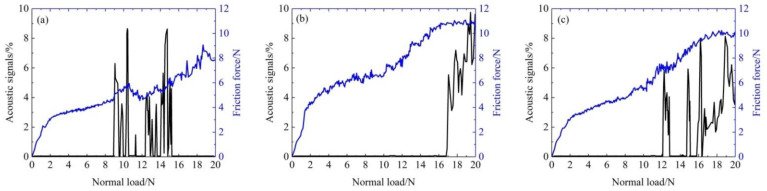
Acoustic signals and the friction force of all composite coatings. (**a**) S2 sample; (**b**) S3 sample; (**c**) S4 sample.

It is generally accepted that the bonding strength of the coating has a favorable effect on its wear resistance and friction reduction performances. The higher the bonding strength of the coating, the better the friction reduction and wear resistance performances [44,45,46]. A way to determine the coating bonding strength by a normal load according to the acoustic signals and friction force was investigated by Li et al. [44] and Chen et al. [47]. To investigate the bonding ability between composite coating and 2218AlA substrate, the critical load is obtained by the scratching method. Figure 8 illustrates the acoustic signals and the friction force of all composite coating samples in scratch experiments. The critical normal load confirms the actual bonding strength of the composite coatings. When the diamond pin is just in contact with the coatings, the acoustic signals are at a steady low value. However, when the acoustic signals fluctuate and sharply improve, it indicates that the composite coating has severe plastic deformation and is peeling off. At this point, the diamond pin contacts the 2218AlA substrate [46]. The critical loads of S2, S3 and S4 samples are 9.07 N, 17.20 N and 12.15 N, respectively. It is found that the critical load of the S3 sample is 1.89 times and 1.42 times that of the S2 and S4 samples, respectively. Thus, the S3 sample has the highest bonding strength. This also illustrates that the S3 sample has superior wear resistance, as shown in Figure 5c.

## 4. Conclusions

(1) The Ni-GO, Ni-SiC and Ni-GO/SiC composite coatings were prepared on the 2218AlA substrate by the electrodeposition technology. The GO or/and SiC reinforcements are uniformly distributed in the composite coatings according to the SEM and EDS results. All composite coatings are dense and compact structures with a good bond to the 2218AlA substrate, and the visible defects and microcracks were not observed at the bonding interface.
(2) Compared with the 2218AlA substrate, the response time, friction coefficient and wear rate of the composite coatings were obviously reduced. The average friction coefficient of Ni-GO, Ni-SiC and Ni-GO/SiC composite coatings were reduced by 54.65%, 35.47% and 50.29%, respectively. However, considering the overall performance, the tribological performances of the Ni-GO/SiC composite coating are superior.
(3) The dominated wear mechanism of the 2218AlA substrate is considered to be a combination of severe adhesive wear, abrasive wear and mild fatigue wear. The main wear mechanism of the Ni-GO composite coating exhibits mild adhesive wear and abrasive wear. Typical abrasive wear and spalling are the dominant wear mechanism of the Ni-SiC composite coating. For the Ni-GO/SiC composite coating, a combination of mild adhesive wear, mild abrasive wear and spalling is supposed as the dominant wear mechanism, indicating the wear characteristics of both Ni-GO composite coating and Ni-SiC composite coating coexist. Still, the damage degree has been significantly reduced.
(4) The microhardness of the Ni-GO, Ni-SiC and Ni-GO/SiC composite coatings is significantly higher than that of the 2218AlA substrate, increased by 121.31%, 214.34% and 178.84%, respectively.

## Figures and Tables

**Table 1:** Chemical composition of 2218AlA substrate.

Elements	Fe	Si	Mn	Cu	Mg	Al
wt.%	0.5	0.35	0.28	1.8	2.6	balanced

**Table 2:** Composition of plating solution and operating conditions.

Composition and Conditions	Parameters		
	Ni-GO Coating	Ni-SiC Coating	Ni-GO/SiC Coating
Ni(NH_2_SO_3_)_2_·4H_2_O (g/L)	450	450	450
HBO_3_ (g/L)	38	38	38
CH_3_(CH_2_)_11_OSO_3_Na (g/L)	0.1	0.1	0.1
SiC (g/L)	0	10	10
GO (g/L)	1	0	1
pH	3	3	3
Stirring rate (rpm)	300	300	300
Temperature (°C)	50	50	50
Current density (A/dm^2^)	5	5	5
Deposition time (min)	60	60	60

## Data Availability

The data presented in this work are available on request from the corresponding authors.
